# The Impact of Socioeconomic Factors and Racial Disparities on Complications in Breast Conservation Surgery: A Single‐Institution Retrospective Cohort Study

**DOI:** 10.1155/tbj/8459253

**Published:** 2026-04-04

**Authors:** Liane N. Obaid, Janice S. Zhang, Grace Y. Lee, Fardeen Bhimani, Susan L. Jao, Carolyn Rachofsky, Lesley G. Coe, Arianna Cavalli, Michelle Ki, Juan Lin, Anjuli Gupta, Sheldon Feldman, Maureen McEvoy

**Affiliations:** ^1^ Department of Surgery, Albert Einstein College of Medicine, Bronx, New York, USA, yu.edu; ^2^ Breast Surgery Division, Department of Surgery, Montefiore Medical Center, Montefiore Einstein Center for Cancer Care, Bronx, New York, USA, montefiore.org

**Keywords:** BCS, breast cancer, breast conservation, complications, ethnic minority

## Abstract

**Background:**

Breast conservation surgery (BCS) is considered to be a low‐morbidity procedure; however, postoperative complications may adversely affect the quality of life of breast cancer survivors. Social determinants of health have previously been shown to impact postoperative outcomes in various other medical conditions. However, there is a paucity of literature that comprehensively examines the social determinants that impact BCS complications or how race influences specific surgical outcomes. This study thus seeks to address this gap by analyzing the impact of these factors within our diverse patient population in the Bronx.

**Methods:**

A retrospective chart review was conducted that examined patients who underwent BCS between January 2016 and December 2022 at a single institution. Patient information, such as comorbidities, Distressed Communities Index (DCI), BMI, race, insurance status, age, pathology, surgery details, adjuvant therapy, and complications, was collected. Univariate and multivariate analyses were utilized to determine the relationship between these variables and postoperative complications.

**Results:**

A total of 627 patients were included in the study. Of these patients, 512 (81.5%) were either Hispanic or African American, 46 patients (7.3%) were White, and 69 (11%) belonged to other races. There was a delay in 37 (6.2%) patients receiving adjuvant therapy. Our study found that higher DCI quintile and higher BMI were associated with a statistically significant increase in overall complication rate (*p* = 0.044, *p* = 0.05, respectively). Race had an association with persistent pain (*p* = 0.003). Former smokers (OR = 3.44, 95% CI [1.419, 8.34], *p* = 0.0062), hypertensive patients (OR = 3.846, 95% CI [1.13, 13.12], *p* = 0.0314), and patients who received adjuvant chemotherapy (OR = 2.756, 95% CI [1.117, 6.801], *p* = 0.0278) had higher odds of developing any complication. Patients who developed any complication were more likely to have a delay in adjuvant therapy than patients who did not have complications (OR = 6.452, CI [2.696, 15.44], *p* < 0.001).

**Conclusion:**

BCS is generally a safe procedure with minimal complications; however, patient‐specific factors are associated with increased complication rates which can lead to delays in adjuvant therapy. Future studies with larger sample sizes are needed to adequately compare outcomes among minority racial groups to a larger reference population.

## 1. Background

Breast cancer is one of the most common cancers among women in the United States, affecting over 300,000 patients in 2025, and is a major contributor to cancer‐related deaths. Treatment is often multimodal, using chemotherapy, radiotherapy, and immunotherapy. However, breast surgery, including breast conservation surgery (BCS) or lumpectomies, is still a mainstay of treatment today [1].

BCS is the standard care for early‐stage breast cancers and is traditionally considered a low‐morbidity procedure with minimal postoperative complications. Nevertheless, when complications do occur, they can significantly impact survivors’ quality of life and may result in delays in adjuvant therapy [2, 3]. Postoperative complications of BCS can include seroma formation, surgical site infection (SSI), hematomas, wound dehiscence, and persistent postoperative pain.

Seromas are fluid collections that arise in the surgical cavity following a lumpectomy and have been thought to be due to an inflammatory response from manipulation of tissue during surgery [2]. Retrospective analyses have identified associations between seroma formation and factors, such as surgery duration, volume of tissue removed, and surgical approach, with mastectomy associated with higher rates of seroma than BCS [3]. Additional studies have also established an association between seroma development and body mass index (BMI) as well as comorbidities, such as diabetes mellitus [4–7].

SSIs occur in a large proportion of breast surgery cases, often reported between 2% and 19%. These cases range widely in severity, from minor and localized to systemic septic infections [2]. Previous studies have identified high BMI, hypertension, advanced age, diabetes, smoking, previous breast operation, and preoperative chemotherapy or radiation as risk factors for SSI [8, 9]. Wound dehiscence, or reopening of previously closed surgical incisions, is another commonly encountered complication, with risk factors including formation of seroma, tissue necrosis at the surgical flap, immediate reconstruction, higher BMI, depression, and tobacco use [10].

Hematoma, or collections of blood at the surgical site, can also account for postoperative complications in BCS and is a common cause for returning to the operating room [11]. Several studies have explored the association of the use of perioperative non–steroidal anti‐inflammatory drugs (NSAIDs) and antithrombotic medication with the incidence of hematoma, finding that the use of NSAIDs does not impact operative or postoperative bleeding, but the use of antithrombotic medication does [12–14]. However, few studies have explored other comorbidities associated with hematoma formation in breast surgery patients.

Persistent postsurgical pain, typically defined clinically by patients as discomfort that lasts greater than 2 months postoperatively without an identifiable underlying cause (such as infection), is a complication that can severely impair the quality of life of breast cancer survivors [15]. A meta‐analysis by Wang et al. demonstrated that younger patients, patients who received radiotherapy, and patients who underwent axillary node dissection had higher incidences of persistent postoperative pain [15]. Additional factors, such as high BMI, larger tumor size, adjuvant chemoradiation, psychological distress, and poor coping mechanisms, have also been implicated [16, 17].

While existing research has established links between postoperative complications and clinical factors, such as comorbidities and surgical techniques, there remains a paucity of literature addressing the role of socioeconomic factors and social determinants of health in shaping the outcomes of BCS [8, 9]. Previous studies have shown that patients from under‐resourced countries often have delays in diagnoses and treatment for breast cancer, resulting in advanced staging at presentation, more complex procedures as treatment, and therefore a higher morbidity and mortality from resultant complications [18]. It has also been previously shown that complications from breast implantation procedures are associated with lower median household income [19, 20]. Lower socioeconomic status was also associated with patients reporting a worse quality of life following breast surgery [21]. Racial disparities have also been documented; one study found that, after adjusting for comorbidities, on average, Black women experienced longer hospitalizations, increased incidences of postoperative complications, and higher in‐hospital mortality than White patients [22].

The Distressed Communities Index (DCI) is a standardized measure that classifies geographic regions by socioeconomic hardship at the zip‐code level based on criteria, such as “high school diploma rate, housing vacancy rate, unemployment rate, poverty rate, and median income ratio.” Communities are graded on a scale of 1–5, with 1 categorized as “*prosperous*” and 5 categorized as “*distressed*” [23]. Although some studies have examined the DCI in relation to breast cancer incidence and mortality, few have explored its association with postoperative complications [24]. The patient population in this study is both racially and socioeconomically diverse, with an average DCI score of 4, presenting a unique opportunity to investigate social determinants of surgical outcomes.

As socioeconomic factors are well‐established in impacting various health outcomes, a more complete picture of how they affect breast surgery complications is necessary. This study thus aims to explore the diverse physical and nonphysical factors that may predispose individuals to adverse outcomes following breast cancer surgery.

## 2. Methods

### 2.1. Study Design

A retrospective chart review of 651 patients who had undergone BCS at Montefiore Medical Center between January 2016 and December 2022 was conducted. The inclusion criteria consisted of (1) age ≥ 18 years, (2) patients who received BCS at our institution, and (3) patients who had at least 3 months of follow‐up at our institution. The exclusion criteria were (1) age ≤ 18 years, (2) pregnant patients, (3) patients who underwent additional procedures, such as mastectomy or breast reconstruction, (4) patients with multifocal or multicentric cancer, and (5) patients who were lost to follow‐up.

### 2.2. Data Collection and Variables

Following the establishment of the inclusion and exclusion criteria, a total of 627 records were retrieved from our institution’s electronic medical record (EMR) system. Patient information was de‐identified and allocated a serial number such that investigators were not able to trace clinical information back to the patients and to minimize bias. The database was kept in an encrypted, password‐protected access file. Demographic data, such as age, BMI, ethnicity, insurance status, and DCI via zip code, were obtained. Information about comorbidities, such as diabetes, hypertension, hyperlipidemia, and smoking status, was also collected. Data pertaining to the patient’s cancer diagnosis and treatment, such as pathology, cancer stage, surgical technique, and neoadjuvant and adjuvant therapy, and postoperative complications (seroma, infection, hematoma, wound dehiscence, and persistent pain), were collected, based on follow‐up notes in the breast clinic. Due to it being scarcely required, analgesic use for the pain was not a documented variable in this study.

### 2.3. Definitions

Postoperative courses were followed for a minimum of 3 months, up to 6 months after the surgery. Complications were identified from clinical documentation by providers in the breast surgery clinic. Seroma was defined clinically as collections of fluid in the surgical cavity, as identified by providers in postoperative visits. Seromas were divided into the subcategories of complicated and uncomplicated seromas, where uncomplicated seromas were managed expectantly and complicated seromas required additional intervention, either with antibiotic treatment or drainage. Postoperative infection was defined as SSI, identified either clinically or with wound culture by clinic providers. Hematoma was defined as collections of blood at the surgical site, identified clinically or with imaging by breast surgery clinic providers. Wound dehiscence was defined as the separation of the wound edges at the closure site as documented in the clinic notes. Persistent pain was defined as pain at the breast for greater than 2 months after the initial operation, without underlying infection or pathology, as reported by the patient and found in postoperative breast‐clinic notes. Delay in adjuvant therapy was defined as a documented delay in initiation of chemotherapy, radiation, or endocrine therapy due to postoperative complications.

### 2.4. Statistical Analysis

Descriptive statistics were employed to characterize the study population, mean or median with standard deviation (SD), or interquartile range (IQR) for continuous variables and frequency (%) for categorical variables. One‐on‐one relationships between demographic characteristics and various comorbid factors with any complication and delay in adjuvant treatment were assessed using the Wilcoxon rank‐sum test or two‐sample *T*‐test for continuous variables and chi‐squared test or Fisher’s exact test when appropriate. Variables with a *p*‐value < 0.20 in bivariate analysis and clinically important factors were considered for inclusion in the following multivariate logistic regression. Multivariate logistic regression included the following variables: presence of complications, age at diagnosis, DCI, race, BMI, smoking status, diabetes, hypertension, hyperlipidemia, surgery type, intraoperative X‐ray radiation therapy with the Intrabeam device, and adjuvant chemotherapy. Smoking status was categorized as “current” if the patient was a current tobacco user, “former” if the patient reported any prior history of smoking, and “never” if the patient denied any history of smoking. Pack‐years were collected but were inconsistently documented in records, so they were omitted from the analysis. Missing data were omitted from the analysis. Quantitative variables, such as age, DCI, and BMI, were treated as continuous variables. A two‐sided *p*‐value < 0.05 was recognized as statistically significant, and odds ratios and their 95% confidence interval (CI) were employed to evaluate strengths of associations. All analyses were conducted using SAS Version 9.4 (SAS Institute Inc.).

## 3. Results

### 3.1. Patient Characteristics

A total of 651 patients who underwent BCS were identified at our institution from January 2016 to December 2022. The mean age of the patients was 63 ± 11.6 years, and the mean BMI was 29.3 ± 6.7 kg/m^2^. The cohort was predominantly composed of ethnic minorities (Hispanic or African American, 83.9%). Most patients had government‐issued insurance, with 132 (21.3%) having private coverage. The mean DCI was 4.14 ± 0.85 (Table 1).

**TABLE 1:** Demographic information.

Variable	Level	*N*	Overall *N* = 621	Any complication *N* = 96	No complication *N* = 525
Age at diagnosis		620	63 (55, 72)	63 (57, 71)	63 (55, 72)

Age at diagnosis	< 65	620	329 (53.06%)	50 (52.63%)	279 (53.14%)
	> 65		291 (46.94%)	45 (47.37%)	246 (46.86%)

Race and ethnicity	African American	568	234 (41.20%)	29 (34.12%)	205 (42.44%)
	Asian		15 (2.64%)	3 (3.53%)	12 (2.48%)
	Hispanic		273 (48.06%)	44 (51.76%)	229 (47.41%)
	White		46 (8.10%)	9 (10.6%)	37 (7.66%)

BMI		621	29.3 (25.7, 33.8)	29.9 (24.6, 34.6)	29.2 (25.7, 33.6)

BMI	Normal	621	125 (20.13%)	25 (26.04%)	100 (19.05%)
	Obese		276 (44.44%)	47 (48.96%)	229 (43.62%)
	Overweight		220 (35.43%)	24 (25.00%)	196 (37.33%)

Employment status	No	620	429 (69.19%)	63 (65.63%)	366 (69.85%)
	Yes		191 (30.81%)	33 (34.38%)	158 (30.15%)

Medicare	No	620	259 (41.77%)	41 (42.71%)	218 (41.60%)
	Yes		361 (58.23%)	55 (57.29%)	306 (58.40%)

Medicaid	No	619	299 (48.30%)	52 (54.17%)	247 (47.23%)
	Yes		320 (51.70%)	44 (45.83%)	276 (52.77%)

Private insurance	No	620	488 (78.71%)	76 (79.17%)	412 (78.63%)
	Yes		132 (21.29%)	20 (20.83%)	112 (21.37%)

Insurance	Medicaid	620	488 (78.71%)	76 (79.17%)	412 (78.63%)
	Private		132 (21.29%)	20 (20.83%)	112 (21.37%)

DCI		620	4 (4, 5)	4 (4, 5)	4 (4, 5)

DCI	< 3	620	33 (5.32%)	4 (4.17%)	29 (5.53%)
	> 3		587 (94.68%)	92 (95.83%)	495 (94.47%)

Smoking status	Active	621	63 (10.1%)	15 (15.6%)	48 (9.14%)
	Former		163 (26.25%)	30 (31.25%)	133 (25.33%)
	Never		395 (63.61%)	51 (53.13%)	344 (65.52%)

Diabetes	No	621	422 (67.95%)	68 (70.83%)	354 (67.43%)
	Yes		199 (32.05%)	28 (29.17%)	171 (32.57%)

Hypertension	No	621	174 (28.02%)	27 (28.13%)	147 (28.00%)
	Yes		447 (71.98%)	69 (71.88%)	378 (72.00%)

Hyperlipidemia	No	621	284 (45.73%)	36 (37.50%)	248 (47.24%)
	Yes		337 (54.27%)	60 (62.50%)	277 (52.76%)

Surgery type	Localized lumpectomy	621	520 (83.74%)	80 (83.33%)	440 (83.81%)
	Lumpectomy		101 (16.26%)	16 (16.67%)	85 (16.19%)

Pathology size		578	14 (8, 22)	13 (8, 22)	14.5 (8, 22)

Neoadjuvant chemotherapy	No	621	561 (90.34%)	87 (90.63%)	474 (90.29%)
	Yes		60 (9.66%)	9 (9.38%)	51 (9.71%)

Neoadjuvant endocrine therapy	No	621	601 (96.78%)	92 (95.83%)	509 (96.95%)
	Yes		20 (3.22%)	4 (4.17%)	16 (3.05%)

Intraoperative radiation therapy	No	621	574 (92.43%)	86 (89.58%)	488 (92.95%)
	Yes		47 (7.57%)	10 (10.42%)	37 (7.05%)

Adjuvant radiation therapy	No	621	178 (28.66%)	32 (33.33%)	146 (27.81%)
	Yes		443 (71.34%)	64 (66.67%)	379 (72.19%)

ER	Negative	566	103 (18.20%)	16 (17.78%)	87 (18.28%)
	Positive		463 (81.80%)	74 (82.22%)	389 (81.72%)

PR	Negative	563	175 (31.08%)	26 (29.21%)	149 (31.43%)
	Positive		388 (68.92%)	63 (70.79%)	325 (68.57%)

Her2	Negative	484	442 (91.32%)	69 (89.61%)	373 (91.65%)
	Positive		42 (8.68%)	8 (10.4%)	34 (8.35%)

Axillary lymph node dissection	No	620	602 (97.10%)	94 (97.92%)	508 (96.95%)
	Yes		18 (2.90%)	2 (2.08%)	16 (3.05%)

Axillary reverse mapping	No	621	541 (87.12%)	82 (85.42%)	459 (87.43%)
	Yes		80 (12.88%)	14 (14.58%)	66 (12.57%)

Sentinel lymph node biopsy	No	620	241 (38.87%)	31 (32.29%)	210 (40.08%)
	Yes		379 (61.13%)	65 (67.71%)	314 (59.92%)

Adjuvant chemotherapy	No	621	515 (82.93%)	81 (84.38%)	434 (82.67%)
	Yes		106 (17.07%)	15 (15.63%)	91 (17.33%)

Adjuvant endocrine therapy	No	621	168 (27.05%)	24 (25.00%)	144 (27.43%)
	Yes		453 (72.95%)	72 (75.00%)	381 (72.57%)

Days from surgery to adjuvant therapy		561	48 (34, 69)	50.5 (40, 69)	48 (33, 69)

*Note:* Demographic information of all variables collected for patients.

Abbreviations: BMI = body mass index, DCI = Distressed Communities Index, ER = estrogen receptor, PR = progesterone receptor.

### 3.2. Tumor Characteristics and Treatment

Of the total patients, 520 (83.7%) underwent seed‐ or wire‐localized lumpectomy, and 101 (16.3%) underwent standard lumpectomy for a palpable mass. Sentinel lymph node biopsy (SLNB) was performed in 379 patients (61.1%). Neoadjuvant therapy was administered to 8 patients (chemotherapy 9.7% and endocrine therapy 3.2%). Postoperatively, 443 patients (71.3%) received radiation therapy (median dose 42.4 Gy), 453 (72.9%) received endocrine therapy, and 106 (17.1%) received adjuvant chemotherapy (Table 1).

Pathology demonstrated ductal carcinoma in situ in 27.1%, invasive ductal carcinoma in 60.1%, invasive lobular carcinoma in 5.5%, and lobular carcinoma in situ or unspecified invasive mammary carcinoma in 7.2% of patients, which had features of both ductal and lobular carcinomas. Most tumors were stage 0–1 (82.1%, Table 2).

**TABLE 2:** Cancer pathology and staging.

Pathology	
DCIS	167 (27.1%)
IDC	371 (60.1%)
ILC	34 (5.50%)
LCIS, IMC	45 (7.20%)
Stages	
0	202 (32.2%)
1	313 (49.9%)
2	80 (12.8%)
3	10 (1.60%)
4	0 (0%)

*Note:* Cancer pathology and staging for all patients. Percentages depicted are out of *N* = 617.

Abbreviations: DCIS = ductal carcinoma in situ, IDC = invasive ductal carcinoma, ILC = invasive lobular carcinoma, IMC = invasive mammary carcinoma, LCIS = lobular carcinoma in situ.

### 3.3. Complications

Ninety‐six patients (15.4%) experienced at least one postoperative complication within 6 months. The most common complications were persistent pain (14.1%), SSI (8.2%), hematoma (4.0%), and wound dehiscence (1.6%) (Table 3). Uncomplicated seromas were excluded from the primary complication outcome; however, 159 patients (25.4%) developed seromas, of whom 86 required intervention and were included as complications. Of these, 68 (42.7%) were drained in office, 16 (10.0%) required antibiotic treatment due to infection, and 2 (1.2%) were evacuated during re‐excision for positive margins (Table 4). Given the frequency of presentation, uncomplicated seroma formation that did not require additional treatment was not included in total complications, as they were extremely common and did not necessarily affect any patient management. This was done to separate the general presence of uncomplicated seroma formation from complicated seromas that required treatment, similar to other complications included.

**TABLE 3:** Postoperative complication frequencies and subtypes.

Complications	
Total	96 (15.4%)
Infection	51 (8.21%)
Hematoma	24 (4.04%)
Wound dehiscence	10 (1.61%)
Persistent pain	88 (14.1%)
Seroma	159 (25.6%)
Delay in adjuvant initiation	37 (6.20%)

*Note:* Complications stratified by type of complication. Percentages out of *N* = 621.

**TABLE 4:** Seroma formation and treatment frequencies.

Seroma	
Total	159 (25.6%)
Drained in clinic	68 (42.7%)
Antibiotic therapy	16 (10.0%)
Evacuated in OR	2 (1.20%)

*Note:* Frequency of seroma formation and treatments. Percentages out of *N* = 159.

On univariate analysis, higher DCI was associated with complications (*p* = 0.044), while higher BMI demonstrated a borderline association (*p* = 0.050) (Table 5). Multivariate logistic regression identified former smoking (OR 3.44; 95% CI [1.42–8.34]; *p* = 0.006), hypertension (OR 3.85; 95% CI [1.13–13.12]; *p* = 0.031), and receipt of adjuvant chemotherapy (OR 2.76; 95% CI [1.12–6.80]; *p* = 0.028) as independent predictors of complications. Race and ethnicity were not significantly associated with overall complication risk (Table 6).

**TABLE 5:** Univariate analysis of complications by risk factors.

Variable	No complication (*N* = 525) (%)	Complication (*N* = 96) (%)	*p*‐value
DCI			
Less than 3	5.50%	4.10%	0.044 (continuous)
Greater or equal to 3	94.4%	95.8%	
Median (Q1, Q3)	4 (4, 5)	4 (4, 5)	
BMI			
Normal	19.0%	26.0%	0.050
Overweight	37.3%	25.0%	
Obese	43.6%	48.9%	

*Note:* *p*‐value for DCI is a continuous variable; *p*‐value for BMI is for stratified variable.

Abbreviations: BMI = body mass index, DCI = Distressed Communities Index.

**TABLE 6:** Significant values in multivariate analysis for any complication.

Risk factor	Odds ratio (OR)	95% confidence interval (CI)	*p*‐value
Former smokers vs. never‐smokers	3.44	1.419–8.340	0.0062
Hypertension vs. normotensive	3.85	1.127–13.12	0.0314
Received adjuvant chemotherapy	2.76	1.117–6.801	0.0278

In subgroup analyses, infections were more common among smokers (*p* = 0.050) and patients undergoing SLNB (*p* = 0.041). Persistent pain varied by race (*p* = 0.003). Seroma formation was associated with hypertension (*p* = 0.018), intraoperative radiation therapy (*p* < 0.001), and SLNB (*p* < 0.001) (Table 7).

**TABLE 7:** Specific postoperative complications by risk factor in univariate analysis.

Complication	Associated factor	No complication (n/N) (%)	Complication (n/N) (%)	*p*‐value
Infection	Sentinel lymph node biopsy	341/570 (59.9%)	38/51 (74.5%)	0.041
	Smoking			
	Active	54/570 (9.50%)	9/51 (17.7%)	0.05
	Former	146/570 (25.6%)	17/51 (33.3%)	
	Never	370/570 (64.9%)	25/51 (49.0%)	

Persistent pain	Race			
	African American	215/533 (44.4%)	19/88 (22.6%)	0.003
	Hispanic	220/533 (45.4%)	53/88 (63.1%)	
	White	37/533 (7.60%)	9/88 (10.7%)	
	Asian	12/533 (2.40%)	3/88 (3.60%)	

Seroma	Hypertension	321/462 (69.5%)	126/159 (79.3%)	0.018
	Intraoperative radiation	25/462 (5.40%)	22/159 (13.8%)	< 0.001
	Sentinel lymph node biopsy	264/462 (57.3%)	115/159 (72.3%)	< 0.001

### 3.4. Delay in Adjuvant Therapy

Among 579 patients receiving adjuvant therapy, 37 (6.3%) experienced delayed initiation (mean of 67 days). Any postoperative complication was strongly associated with treatment delay (OR 6.45; 95% CI [2.70–15.44]; *p* < 0.001). Hypertension (OR 3.69; 95% CI [1.05–12.94]; *p* = 0.041) and former smoking (OR 3.27; 95% CI [1.31–8.13]; *p* = 0.011) were also independently associated with delays (Table 8).

**TABLE 8:** Significant values in multivariate analysis for delay in adjuvant treatment.

Risk factor	Odds ratio (OR)	95% confidence interval (CI)	*p*‐value
Any complication vs. none	6.452	2.696–15.44	< 0.001
Hypertension vs. normotensive	3.690	1.052–12.94	0.0414
Former smokers vs. never‐smokers	3.267	1.313–8.133	0.0109

## 4. Discussion

BCS is typically considered a low‐morbidity procedure and remains a key treatment for early‐stage breast cancer. However, complications can arise that impact quality of life, prolong hospital stays, require reoperation, and ultimately delay adjuvant therapy. This study further examines several clinical and demographic factors associated with these postoperative complications.

This study found that increased DCI was associated with a statistically significant increase in the occurrence of any complication (*p* = 0.044) on univariate analysis. While this association was not observed in multivariate regression, this does not negate the potential impact of DCI on postoperative complications, as many factors explaining how community distress influences outcomes may have been accounted for in the multivariate model. The multivariate model provides independent effect estimates; however, it is important to note that the factors that the multivariate model adjusts for confound the DCI results, due to the natural association of those factors with lower socioeconomic status. When controlling for factors, such as BMI, hypertension, diabetes, and smoking status (that all have higher incidences in more under‐resourced populations), it is understandable why DCI as an independent factor would not stand alone in multivariate analysis.

Numerous studies have demonstrated the impact of community distress on medical outcomes. Elevated DCI scores have been shown to correlate with increased incidences of hospital readmission, repeated surgical intervention, and surgical site complications in ventral hernia repairs and infra‐inguinal bypass procedures [25, 26]. Patients from under‐resourced areas have been shown to typically present with further progressed stages of disease, have a higher burden of comorbidities, and are more likely to have adverse lifestyle factors, such as smoking, leading to higher short‐term and long‐term mortality [27, 28]. Martin et al. showed in their study that socioeconomic status was specifically correlated with higher rates of postoperative infection in breast implantation [19]. Sarver et al. additionally demonstrated that patients with government insurance, such as Medicaid, had a higher rate of postoperative complications, though our study did not find a similar association [29]. Though these studies are not focused on BCS specifically, they can give insight into the socioeconomic factors that may impact patients who receive general breast surgery. Additional studies evaluating factors, such as distance to a hospital, trust in the healthcare system, access to providers of similar cultural and racial backgrounds, and language barriers, can potentially shed more light on the association of DCI with operative outcomes.

Although some studies have not shown better survival rates in more resourced communities, this does not preclude the possibility of poorer outcomes in distressed communities [30]. Notably, Herbert et al. demonstrated that, on a percentile scale, a 10‐point decrease in DCI was associated with a rise in the proportion of women who received an age‐appropriate mammogram screening. This result suggested that patients in more affluent areas were more likely to receive adequate screening. This study also demonstrated higher detection rates and lower mortality in the same population, showing the efficacy of screening mammograms in the prevention of poor health outcomes [24]. In cancers with established screening programs, such as breast cancer, this study provides valuable insight into economic and social barriers that can contribute to adverse outcomes in our patient population and can inform allocation of resources. It demonstrates that there is an opportunity for community intervention in the treatment of patients that come from lower socioeconomic communities.

While our patient population consists of more diversity compared to the national reference population, this study did demonstrate a statistically significant difference in persistent pain between races (*p* = 0.003), where out of 88 of those who had persistent postoperative pain, 19 (21.3%) identified as African American, 53 (60%) identified as Hispanic, 9 (10.7%) were White, and 3 (3.57%) were Asian. This complication was found to appreciably impact postoperative quality of life in our patient population. The pain could have also been due to postoperative radiation, incision placement, and scar tissue, all of which were not accounted for in this analysis. Previous literature has shown that BCS (as opposed to mastectomy), radiotherapy, and younger age are associated with higher incidences of persistent pain, but few have explored the impact of race and ethnicity on breast surgery pain outcomes [31, 32]. Our study did not find a correlation between race and any other complications, nor was there a correlation in multivariate analysis; however, the presence of a correlation with persistent pain is notable. Though not robust, the findings of this study contribute to the established body of literature addressing the impact of race and ethnicity on medical outcomes. After adjusting for comorbid conditions, other studies have shown that Black patients still have an elevated risk of in‐hospital mortality, greater incidence of postoperative and chemotherapy‐related complications, more frequent flap necrosis, and worse prognostic outcomes compared to White patients [22, 33–36]. Further, these findings corroborate previous studies that have shown racial disparities in the experience of pain following surgery, including Sadhasivam et al. who showed that African American pediatric patients experienced more pain following tonsillectomy than White patients [37]. Lillis et al. also found that pain intensity variations were influenced by socioeconomic disparities linked to race and ethnicity, using structural equation modeling [37–39]. Our study did not have a large enough reference population to draw robust conclusions about the impact of race and ethnicity like the aforementioned studies. Therefore, further studies in comparison with a national reference population to elucidate how ethnicity may affect persistent pain following BCS are necessary. Regardless of the etiology of the increased pain (whether it is biological or sociological), these findings can direct interventions in ethnically diverse populations to minimize postoperative pain, as well as other adverse outcomes, as much as possible.

Smoking has been consistently linked to adverse surgical outcomes, including increased risks of infections. In our study, former smokers were found to have 3.44 times greater odds of experiencing any complication compared to never‐smokers, while both active and former smokers demonstrated an overall higher probability of developing complications (*p* = 0.0214). This aligns with prior research showing that tobacco use disrupts collagen synthesis, suppresses inflammatory response, and constricts vessels that limit tissue oxygenation, resulting in hindered wound healing and prolonging the recovery process [40]. These findings underscore the importance of preoperative smoking cessation to mitigate surgical risks.

Patients with hypertension had 3.846 times higher odds of developing any postoperative complication compared to normotensive patients. This finding is consistent with other surgical literature, including a study in total hip arthroplasty, where hypertension has been associated with impaired healing and lengthened recovery time, highlighting its broader impact on postoperative outcomes [41]. Complications in our cohort were likely influenced by microvascular compromise and subsequent decreased tissue oxygenation, which may have disrupted effective healing.

Patients who received adjuvant chemotherapy in our study had 2.756 times higher odds of developing any complication (*p* = 0.0278). In support of this, though the difference was not statistically significant, a retrospective study of 112 patients found that patients who had mastectomy with breast reconstruction and adjuvant chemotherapy had an increased rate of surgical site complications compared to those who did not receive adjuvant chemotherapy [42]. Another retrospective analysis of 163 patients who underwent mastectomy and immediate breast reconstruction found no statistically significant difference in overall complication rates among those who received neoadjuvant or adjuvant chemotherapy compared to those who did not undergo chemotherapy at all. Within this study, 18 of the 41 (44%) patients who had adjuvant chemotherapy, 13 (23%) of the patients in the neoadjuvant group, and 16 (25%) in the nonchemotherapy group developed SSIs postoperatively. Despite the adjuvant group having the highest incidence of postoperative infections, the study concluded that whether a patient received neoadjuvant or adjuvant chemotherapy did not have a bearing on the overall rate of complications after mastectomy and reconstruction [43]. A prospective observational multicenter study found a link between lower socioeconomic status and the administration of reduced doses of adjuvant chemotherapy in breast cancer patients, which could potentially increase the risk of postoperative complications due to suboptimal dosing [44]. Further research is needed to clarify the association of neoadjuvant therapy with postoperative complications to optimize postoperative management in breast cancer patients.

Our data demonstrated a borderline statistically significant association (*p* = 0.050) between higher BMI and the occurrence of complications following breast‐conserving surgery. This finding aligns with existing evidence, such as the multicenter analysis by De La Cruz Ku et al. that evaluated 6887 patients and found that those with a BMI of 35 or greater who had BCS had an elevated risk of wound dehiscence and postoperative infections [45]. Given that lower socioeconomic status is often associated with higher rates of obesity, these findings reinforce the need to address social determinants of health to mitigate surgical risks.

Seroma formation in our cohort was significantly associated with intraoperative radiation therapy (*p* < 0.001), SLNB (*p* < 0.001), and hypertension (*p* = 0.018) in our cohort, suggesting that multiple factors may contribute to its development. In a retrospective review of 667 women who underwent BCS and SLNB, 19% of whom had axillary seromas, found diabetes and tobacco use, but not hypertension, to be significantly associated with seroma formation [46]. Another retrospective study identified hypertension as a statistically significant predictor for seroma following mastectomy, suggesting that elevated blood pressures may increase the likelihood of seromas through persistent wound drainage [47]. These inconsistencies highlight the multifactorial nature of seroma development and the need for further studies to better elucidate the impact of specific comorbidities on its occurrence. Intraoperative radiation therapy has been shown in one retrospective cohort study to be associated with higher incidence of seroma formation, wound infections, and prolonged healing compared to patients who underwent BCS alone [48]. Although our study primarily focuses on socioeconomic factors in breast‐conserving surgery, our findings suggest that intraoperative radiation therapy, comorbidities, and surgical technique play a role in seroma formation, highlighting the need for vigilant postoperative monitoring.

The standard treatment pathway for breast cancer involving BCS typically includes surgery followed by adjuvant therapy, such as radiation, chemotherapy, and/or endocrine therapy. In our study, we found a strong correlation between postoperative complications and delayed adjuvant therapy initiation: Patients who experienced any complication were 6.45 times more likely to have a delay compared to those without complications (OR 6.452; 95% CI: 2.696–15.44; *p* < 0.001). A delay in adjuvant therapy has the potential to impact outcomes of cancer progression, as demonstrated by Trufelli et al. who showed in their study that the mortality risk increased by 1.3‐fold for every month of delay in initiating adjuvant treatment following breast cancer surgery [49]. Several other studies have also shown that postponing adjuvant therapy is linked to an increased risk of cancer‐related death, regardless of other contributing factors [49–51].

Both hypertension and a history of smoking significantly increased the likelihood of delays in adjuvant therapy. Hypertensive patients were 3.69 times more prone to these delays, while former smokers were 3.27 times more likely to experience them compared to never‐smokers. While several studies discuss the impact of smoking and hypertension on cancer outcomes, data that explicitly focus on these factors correlating with delays in initiating therapy are limited. Our findings suggest that these common comorbidities may be associated with treatment delays more than previously recognized. Prior studies have identified statistically significant associations between delayed initiation of chemotherapy and low socioeconomic status, identifying as non‐Hispanic Black and Hispanic ethnicities, and enrollment in Medicare or Medicaid insurance coverage [52]. Our results underscore the importance of addressing health disparities, managing hypertension, and counseling on smoking cessation to minimize potential delays in adjuvant chemotherapy.

In the context of previous studies showing increased adverse outcomes, specifically mortality, associated with such a delay, coupled with the context of the socioeconomic factors correlating with the rate of these complications, these findings suggest that individuals in more distressed communities, or with lower socioeconomic status, or of an ethnic minority, may be correlated with a higher risk in terms of cancer progression. Thus, survival outcomes may be impacted by initiatives to minimize the gap between surgery and initiation of adjuvant therapy. Future studies should focus on evaluating the outcomes of patients who experienced delays in adjuvant therapy and should further investigate the role of racial disparities and specific social determinants of health in contributing to complications that may exacerbate these delays.

### 4.1. Limitations

The sample size of our study was relatively small, which may have limited the ability to identify statistically significant trends or associations, particularly in subgroup analyses due to lack of power. Our study is also limited by the follow‐up data available in the EMR, as treatment details may be affected by documentation discrepancies and incomplete follow‐up appointments. Third, the retrospective design of our study may introduce bias and limitations in data accuracy and follow‐up. Our study exclusively draws data from a single institution, which may limit the generalizability of the findings to broader populations and medical settings. Further studies using a matched control group would create more generalizability, however under the framework of this study with this patient population was deemed unfeasible. Other important confounders unaccounted for include breast volume, duration of surgery, surgeon variability, time from smoking cessation to cancer diagnosis, and psychosocial comorbidities. These factors may play a role in the development of complications but were not included in the analysis in this study. Finally, this study omitted patients who were lost to follow‐up due to lack of sufficient data for analysis. Though the patients transferred care out of state, the omission of these patients may have changed the results of the study.

## 5. Conclusion

Our study found that DCI and BMI were associated with a statistically significant difference in overall complication rate in individual comparison, though these results were not reflected in multivariate analysis. Race and ethnicity had an association with persistent pain. Patients who developed any complication were statistically more likely to have a delay in adjuvant therapy than patients who did not have complications.

## Author Contributions

Maureen McEvoy, Sheldon Feldman, and Anjuli Gupta supervised the study. Fardeen Bhimani, Susan L. Jao, and Michelle Ki contributed to manuscript revision and quality control. Juan Lin contributed significantly to statistical analyses. Janice S. Zhang, Grace Y. Lee, Carolyn Rachofsky, Lesley G. Coe, and Arianna Cavalli contributed to data collection. Liane N. Obaid was responsible for study design, data collection, analysis, and manuscript writing.

## Funding

The author(s) received no financial support for the research, authorship, and/or publication of this article.

## Ethics Statement

This study was approved by the Institutional Review Board at Montefiore Medical Center and Albert Einstein College of Medicine. Written informed consent was not required from patients for publication of this article.

## Conflicts of Interest

The author(s) declare no conflicts of interest.

## Data Availability

Due to privacy regulations and institutional policies, these data cannot be made publicly available. De‐identified data may be available from the corresponding author upon reasonable request and with approval from the Institutional Review Board (IRB).

## References

[1] Siegel R. L. , Kratzer T. B. , Giaquinto A. N. , Sung H. , and Jemal A. , Cancer Statistics, CA: A Cancer Journal for Clinicians. (2025) 75, no. 1, 10–45, 10.3322/caac.21871. 39817679 PMC11745215

[2] Al-Hilli Z. and Wilkerson A. , Breast Surgery: Management of Postoperative Complications Following Operations for Breast Cancer, Surgical Clinics of North America. (2021) 101, no. 5, 845–863, 10.1016/j.suc.2021.06.014. 34537147

[3] Ebner F. , Friedl T. W. P. , de Gregorio A. et al., Seroma in Breast Surgery: All the Surgeons Fault?, Archives of Gynecology and Obstetrics. (2018) 298, no. 5, 951–959, 10.1007/s00404-018-4880-8, 2-s2.0-85053047414. 30196358

[4] Unger J. , Rutkowski R. , Kohlmann T. , Paepke S. , Zygmunt M. , and Ohlinger R. , Potential Risk Factors Influencing the Formation of Postoperative Seroma After Breast Surgery-A Prospective Study, Anticancer Research. (2021) 41, no. 2, 859–867, 10.21873/anticanres.14838. 33517291

[5] Kuroi K. , Shimozuma K. , Taguchi T. et al., Evidence-Based Risk Factors for Seroma Formation in Breast Surgery, Japanese Journal of Clinical Oncology. (2006) 36, no. 4, 197–206, 10.1093/jjco/hyl019, 2-s2.0-33745728715. 16684859

[6] Porter K. A. , O’Connor S. , Rimm E. , and Lopez M. , Electrocautery as a Factor in Seroma Formation Following Mastectomy, Americas Journal of Surgery. (1998) 176, no. 1, 8–11, 10.1016/s0002-9610(98)00093-2, 2-s2.0-0032127662. 9683123

[7] Zielinski J. , Jaworski R. , Irga N. , Kruszewski J. W. , and Jaskiewicz J. , Analysis of Selected Factors Influencing Seroma Formation in Breast Cancer Patients Undergoing Mastectomy, Archives of Medical Science. (2013) 9, no. 1, 86–92, 10.5114/aoms.2012.29219, 2-s2.0-84874855235. 23515419 PMC3598126

[8] Mak J. C. and Kwong A. , Complications in Post-Mastectomy Immediate Breast Reconstruction: A Ten-Year Analysis of Outcomes, Clinical Breast Cancer. (2020) 20, no. 5, 402–407, 10.1016/j.clbc.2019.12.002. 32665188

[9] Xue D. Q. , Qian C. , Yang L. , and Wang X. F. , Risk Factors for Surgical Site Infections After Breast Surgery: A Systematic Review and Meta-Analysis, European Journal of Surgical Oncology. (2012) 38, no. 5, 375–381, 10.1016/j.ejso.2012.02.179, 2-s2.0-84862831094. 22421530

[10] Nickel K. B. , Myckatyn T. M. , Lee C. N. , Fraser V. J. , Olsen M. A. , and Program C. D. C. P. E. , Individualized Risk Prediction Tool for Serious Wound Complications After Mastectomy With and Without Immediate Reconstruction, Annals of Surgical Oncology. (2022) 29, no. 12, 7751–7764, 10.1245/s10434-022-12110-1. 35831524 PMC9937777

[11] Al-Hilli Z. , Thomsen K. M. , Habermann E. B. , Jakub J. W. , and Boughey J. C. , Reoperation for Complications After Lumpectomy and Mastectomy for Breast Cancer From the 2012 National Surgical Quality Improvement Program (ACS-NSQIP), Annals of Surgical Oncology. (2015) 22, no. Suppl 3, S459–S469, 10.1245/s10434-015-4741-7, 2-s2.0-84952875752. 26208580

[12] Tamminen A. , Aaltonen R. I. , and Ristola M. T. , Postoperative Bleeding Complications in Breast Conserving Surgery and the Role of Antithrombotic Medications: Retrospective Analysis of 4712 Operations, World Journal of Surgical Oncology. (2024) 22, no. 1, 10.1186/s12957-024-03511-5. PMC11375840 39232775

[13] Bongiovanni T. , Lancaster E. , Ledesma Y. et al., Systematic Review and Meta-Analysis of the Association Between Non-Steroidal Anti-Inflammatory Drugs and Operative Bleeding in the Perioperative Period, Journal of the American College of Surgeons. (2021) 232, no. 5, 765–90 e1, 10.1016/j.jamcollsurg.2021.01.005. 33515678 PMC9281566

[14] Mikhaylov Y. , Weinstein B. , Schrank T. P. et al., Ketorolac and Hematoma Incidence in Postmastectomy Implant-Based Breast Reconstruction, Annals of Plastic Surgery. (2018) 80, no. 5, 472–474, 10.1097/sap.0000000000001409, 2-s2.0-85045915635. 29538000

[15] Wang L. , Guyatt G. H. , Kennedy S. A. et al., Predictors of Persistent Pain After Breast Cancer Surgery: A Systematic Review and Meta-Analysis of Observational Studies, Canadian Medical Association Journal. (2016) 188, no. 14, E352–E361, 10.1503/cmaj.151276, 2-s2.0-84990042020. 27402075 PMC5047835

[16] Carpenter J. S. , Andrykowski M. A. , Sloan P. et al., Postmastectomy/Postlumpectomy Pain in Breast Cancer Survivors, Journal of Clinical Epidemiology. (1998) 51, no. 12, 1285–1292, 10.1016/s0895-4356(98)00121-8, 2-s2.0-0032446969. 10086821

[17] Alves Nogueira Fabro E. , Bergmann A. , do Amaral E. S. B. et al., Post-Mastectomy Pain Syndrome: Incidence and Risks, Breast. (2012) 21, no. 3, 321–325, 10.1016/j.breast.2012.01.019, 2-s2.0-84862511489. 22377590

[18] GlobalSurg C. and National Institute for Health Research Global Health Research Unit on Global S , Global Variation in Postoperative Mortality and Complications After Cancer Surgery: A Multicentre, Prospective Cohort Study in 82 Countries, Lancet. (2021) 397, no. 10272, 387–397. 33485461 10.1016/S0140-6736(21)00001-5 PMC7846817

[19] Martin M. S. , Kebede S. , Saad O. A. , Baker N. F. , and Losken A. , Impact of Socioeconomic Status on Breast Reconstruction Outcomes, Annals of Plastic Surgery. (2022) 88, no. 5 Suppl 5, S481–S484, 10.1097/sap.0000000000003124. 35276707

[20] Jeevan R. , Browne J. P. , Pereira J. et al., Socioeconomic Deprivation and Inpatient Complication Rates Following Mastectomy and Breast Reconstruction Surgery, British Journal of Surgery. (2015) 102, no. 9, 1064–1070, 10.1002/bjs.9847, 2-s2.0-84937024524. 26075654

[21] Le N. K. , Gabrick K. S. , Chouairi F. , Mets E. J. , Avraham T. , and Alperovich M. , Impact of Socioeconomic Status on Psychological Functioning in Survivorship Following Breast Cancer and Reconstruction, Breast Journal. (2020) 26, no. 9, 1695–1701, 10.1111/tbj.13849. 32337778

[22] Akinyemiju T. F. , Vin-Raviv N. , Chavez-Yenter D. , Zhao X. , and Budhwani H. , Race/Ethnicity and Socio-Economic Differences in Breast Cancer Surgery Outcomes, Cancer Epidemiol. (2015) 39, no. 5, 745–751, 10.1016/j.canep.2015.07.010, 2-s2.0-84941738062. 26231096

[23] Economic Innovation Group Distressed Communities Index, https://eig.org/dci.

[24] Herbert C. , Paro A. , Diaz A. , and Pawlik T. M. , Association of Community Economic Distress and Breast and Colorectal Cancer Screening, Incidence, and Mortality Rates Among US Counties, Annals of Surgical Oncology. (2022) 29, no. 2, 837–848, 10.1245/s10434-021-10849-7. 34585297

[25] Maskal S. M. , Chang J. H. , Ellis R. C. et al., Distressed Community Index as a Predictor of Presentation and Postoperative Outcomes in Ventral Hernia Repair, Americas Journal of Surgery. (2023) 226, no. 5, 580–585, 10.1016/j.amjsurg.2023.06.015. 37331908

[26] Hawkins R. B. , Charles E. J. , Mehaffey J. H. et al., Socioeconomic Distressed Communities Index Associated With Worse Limb-Related Outcomes After Infrainguinal Bypass, Journal of Vascular Surgery. (2019) 70, no. 3, 786–94 e2, 10.1016/j.jvs.2018.10.123, 2-s2.0-85067199247. 31204218

[27] Mehaffey J. H. , Hawkins R. B. , Charles E. J. et al., Socioeconomic “Distressed Communities Index” Improves Surgical Risk-Adjustment, Annals of Surgery. (2020) 271, no. 3, 470–474, 10.1097/sla.0000000000002997. 30741732

[28] Mentias A. , Desai M. Y. , Vaughan-Sarrazin M. S. et al., Community-Level Economic Distress, Race, and Risk of Adverse Outcomes After Heart Failure Hospitalization Among Medicare Beneficiaries, Circulation. (2022) 145, no. 2, 110–121, 10.1161/circulationaha.121.057756. 34743555 PMC9172990

[29] Sarver M. M. , Rames J. D. , Ren Y. et al., Racial and Ethnic Disparities in Surgical Outcomes After Postmastectomy Breast Reconstruction, Journal of the American College of Surgeons. (2022) 234, no. 5, 760–771, 10.1097/xcs.0000000000000143. 35426388 PMC9347225

[30] Nemesure B. , Mermelstein L. K. , and Scarbrough K. H. , Does Community Affluence Improve Survival of Colorectal Cancer?, AJ Focus. (2023) 2, no. 4, 10.1016/j.focus.2023.100144. PMC10628652 37941822

[31] Tasmuth T. , Kataja M. , Blomqvist C. , von Smitten K. , and Kalso E. , Treatment-Related Factors Predisposing to Chronic Pain in Patients With Breast Cancer--A Multivariate Approach, Acta Oncologica. (1997) 36, no. 6, 625–630, 10.3109/02841869709001326, 2-s2.0-0030807768. 9408154

[32] Tasmuth T. , von Smitten K. , Hietanen P. , Kataja M. , and Kalso E. , Pain and Other Symptoms After Different Treatment Modalities of Breast Cancer, Annals of Oncology. (1995) 6, no. 5, 453–459, 10.1093/oxfordjournals.annonc.a059215, 2-s2.0-0029003847. 7669710

[33] Stabellini N. , Cullen J. , Cao L. et al., Racial Disparities in Breast Cancer Treatment Patterns and Treatment Related Adverse Events, Scientific Reports. (2023) 13, no. 1, 10.1038/s41598-023-27578-4. PMC9868122 36683066

[34] Akinyemiju T. , Sakhuja S. , and Vin-Raviv N. , Racial and Socio-Economic Disparities in Breast Cancer Hospitalization Outcomes by Insurance Status, Cancer Epidemiol. (2016) 43, 63–69, 10.1016/j.canep.2016.06.011, 2-s2.0-84977623080. 27394678 PMC5321053

[35] Wang S. , Tang W. , Wang S. , Hong S. , and Liu J. , Racial Disparities in Survival of Breast Cancer Patients After Surgery, Frontiers in Public Health. (2022) 10, 10.3389/fpubh.2022.831906. PMC9136217 35646795

[36] Mets E. J. , Chouairi F. K. , Gabrick K. S. , Avraham T. , and Alperovich M. , Persistent Disparities in Breast Cancer Surgical Outcomes Among Hispanic and African American Patients, European Journal of Surgical Oncology. (2019) 45, no. 4, 584–590, 10.1016/j.ejso.2019.01.016, 2-s2.0-85060237021. 30683449

[37] Sadhasivam S. , Chidambaran V. , Ngamprasertwong P. et al., Race and Unequal Burden of Perioperative Pain and Opioid Related Adverse Effects in Children, Pediatrics. (2012) 129, no. 5, 832–838, 10.1542/peds.2011-2607, 2-s2.0-84860573467. 22529273 PMC3340593

[38] Lillis T. A. , Burns J. , Aranda F. et al., Race-Related Differences in Acute Pain Complaints Among Inner-City Women: The Role of Socioeconomic Status, Journal of Behavioral Medicine. (2020) 43, no. 5, 791–806, 10.1007/s10865-019-00123-3. 31832845 PMC7289669

[39] Bhattacharyya N. and Shapiro N. L. , Associations Between Socioeconomic Status and Race With Complications After Tonsillectomy in Children, Otolaryngology–Head and Neck Surgery. (2014) 151, no. 6, 1055–1060, 10.1177/0194599814552647, 2-s2.0-84914163609. 25301786

[40] Sorensen L. T. , Wound Healing and Infection in Surgery. The Clinical Impact of Smoking and Smoking Cessation: A Systematic Review and Meta-Analysis, Archives of Surgery. (2012) 147, no. 4, 373–383, 10.1001/archsurg.2012.5, 2-s2.0-84859977167. 22508785

[41] Ahmed A. A. , Mooar P. A. , Kleiner M. , Torg J. S. , and Miyamoto C. T. , Hypertensive Patients Show Delayed Wound Healing Following Total Hip Arthroplasty, PLoS One. (2011) 6, no. 8, 10.1371/journal.pone.0023224, 2-s2.0-80051629621. PMC3154930 21853091

[42] Furey P. C. , Macgillivray D. C. , Castiglione C. L. , and Allen L. , Wound Complications in Patients Receiving Adjuvant Chemotherapy After Mastectomy and Immediate Breast Reconstruction for Breast Cancer, Journal of Surgical Oncology. (1994) 55, no. 3, 194–197, 10.1002/jso.2930550313, 2-s2.0-0028220363. 8176932

[43] Warren Peled A. , Itakura K. , Foster R. D. et al., Impact of Chemotherapy on Postoperative Complications After Mastectomy and Immediate Breast Reconstruction, Archives of Surgery. (2010) 145, no. 9, 880–885, 10.1001/archsurg.2010.163, 2-s2.0-77957038318. 20855759

[44] Griggs J. J. , Culakova E. , Sorbero M. E. et al., Effect of Patient Socioeconomic Status and Body Mass Index on the Quality of Breast Cancer Adjuvant Chemotherapy, Journal of Clinical Oncology. (2007) 25, no. 3, 277–284, 10.1200/jco.2006.08.3063, 2-s2.0-33846933756. 17159190

[45] De La Cruz Ku G. , Camarlinghi M. , Mallouh M. P. et al., The Impact of Body Mass Index on Oncoplastic Breast Surgery: A Multicenter Analysis, Journal of Surgical Oncology. (2023) 128, no. 7, 1052–1063, 10.1002/jso.27397. 37448232

[46] Gunn J. , Gibson T. , Li Z. , Diehl N. , Bagaria S. , and McLaughlin S. , Symptomatic Axillary Seroma After Sentinel Lymph Node Biopsy: Incidence and Treatment, Annals of Surgical Oncology. (2016) 23, no. 10, 3347–3353, 10.1245/s10434-016-5398-6, 2-s2.0-84978148837. 27393569

[47] Kumar S. , Lal B. , and Misra M. C. , Post-Mastectomy Seroma: A New Look Into the Aetiology of an Old Problem, Journal of the Royal College of Surgeons of Edinburgh. (1995) 40, no. 5, 292–294. 8523301

[48] Gulcelik M. A. , Dogan L. , Karaman N. et al., Intraoperative Boost Radiation Effects on Early Wound Complications in Breast Cancer Patients Undergoing Breast-Conserving Surgery, Turkish Journal of Medical Sciences. (2017) 47, no. 4, 1185–1190, 10.3906/sag-1605-48, 2-s2.0-85029533980. 29156861

[49] Trufelli D. C. , Matos L. L. , Santi P. X. , and Del Giglio A. , Adjuvant Treatment Delay in Breast Cancer Patients, Revista da Associação Médica Brasileira. (2015) 61, no. 5, 411–416, 10.1590/1806-9282.61.05.411, 2-s2.0-84948686151. 26603003

[50] Hanna T. P. , King W. D. , Thibodeau S. et al., Mortality due to Cancer Treatment Delay: Systematic Review and Meta-Analysis, BMJ. (2020) 371, 10.1136/bmj.m4087. PMC7610021 33148535

[51] Lesage M. , Pilloy J. , Fleurier C. et al., [Prognosis Impact of Breast Cancer Adjuvant Radiotherapy Delay], Gynecol Obstet Fertil Senol. (2019) 47, no. 6, 516–521, 10.1016/j.gofs.2019.03.001, 2-s2.0-85063064738. 30851415

[52] Chavez-MacGregor M. , Clarke C. A. , Lichtensztajn D. Y. , and Giordano S. H. , Delayed Initiation of Adjuvant Chemotherapy Among Patients With Breast Cancer, JAMA Oncology. (2016) 2, no. 3, 322–329, 10.1001/jamaoncol.2015.3856, 2-s2.0-84979503571. 26659132 PMC5920529

